# Dihydroorotate Dehydrogenase in Mitochondrial Ferroptosis and Cancer Therapy

**DOI:** 10.3390/cells14231889

**Published:** 2025-11-28

**Authors:** Jaewang Lee, Jong-Lyel Roh

**Affiliations:** 1 Department of Otorhinolaryngology-Head and Neck Surgery, CHA Bundang Medical Center, CHA University, Seongnam 13496, Republic of Korea; 2 Logsynk, Seoul 06175, Republic of Korea; 3 Department of Biomedical Science, General Graduate School, CHA University, Pocheon 11160, Republic of Korea

**Keywords:** dihydroorotate dehydrogenase, ferroptosis, cancer therapy, pyrimidine metabolism, immunotherapy

## Abstract

**Highlights:**

**What are the main findings?**

- DHODH connects pyrimidine synthesis with mitochondrial ferroptosis defense.
- Elevated DHODH expression promotes tumor growth and resistance.
- DHODH inhibition synergizes with GPX4 blockade to induce ferroptosis.

**What are the implications of the main findings?**

- Targeting DHODH represents a promising strategy to overcome cancer resistance.
- The combination of nanomedicine or immunotherapy enhances therapeutic efficacy.
- Integrating DHODH with lipid and iron metabolism pathways may guide precision therapy.

**Abstract:**

Ferroptosis is an iron-dependent form of regulated cell death driven by lipid peroxidation. Since the identification of dihydroorotate dehydrogenase (DHODH) as a mitochondrial suppressor of ferroptosis in 2021, increasing evidence has highlighted its role in linking nucleotide metabolism, redox regulation, and tumor progression. We conducted a comprehensive review of publications on DHODH, ferroptosis, and cancer. Relevant studies were analyzed to synthesize mechanistic insights, translational implications, and therapeutic perspectives. DHODH, a flavin-dependent mitochondrial enzyme catalyzing the oxidation of dihydroorotate to orotate, integrates pyrimidine biosynthesis with electron transport chain activity. Beyond its canonical metabolic role, DHODH regenerates ubiquinol (CoQ_10_H_2_) to suppress mitochondrial lipid peroxidation and ferroptosis. Elevated DHODH expression in colorectal, hepatocellular, breast, renal, and brain cancers correlates with poor prognosis, therapy resistance, and immune evasion. Pharmacological inhibition of DHODH disrupts pyrimidine synthesis and redox defense, sensitizing GPX4-low tumors to ferroptosis. Preclinical studies demonstrate synergy between DHODH inhibitors and chemotherapy, radiotherapy, or immune checkpoint blockade. Nanoparticle-based delivery systems enhance therapeutic efficacy by simultaneously targeting multiple ferroptosis defense arms while reducing toxicity. DHODH serves as both a metabolic and redox checkpoint in cancer, linking ferroptosis suppression to proliferation and immune escape. Targeting DHODH offers a promising strategy to dismantle cancer resilience, particularly in combination with ferroptosis inducers and immunotherapies. Future research should focus on biomarker-guided stratification, nanomedicine platforms, and clinical translation of DHODH inhibitors.

## 1. Introduction

**Figure 1:**
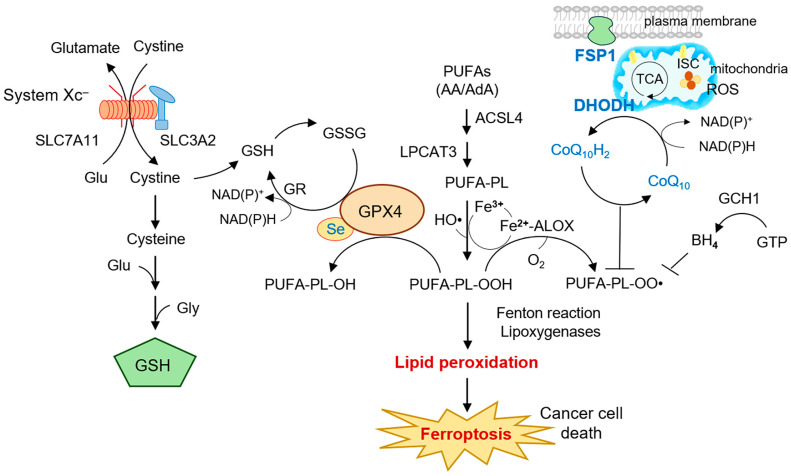
Compartmentalized regulation of ferroptosis by lipid peroxidation and antioxidant defenses. System Xc^−^ (SLC7A11 with SLC3A2) exchanges extracellular cystine for intracellular glutamate; imported cystine is reduced to cysteine and, together with glutamate and glycine, supports glutathione (GSH) synthesis. GSH is recycled from GSSG by glutathione reductase (GR) using NAD(P)H. Arachidonic (AA) and adrenic acid (AdA) are esterified into membrane phospholipids by ACSL4 and LPCAT3 to form PUFA-PLs, which are oxidized by the labile Fe^2+^ pool (Fenton chemistry) and lipoxygenases (ALOX12/15) to generate phospholipid hydroperoxides (PUFA-PL-OOH) and peroxyl radicals (PUFA-PL-OO•). The selenoenzyme GPX4 uses GSH (converting GSH→GSSG) to reduce PUFA-PL-OOH to PUFA-PL-OH, preventing membrane damage. In parallel, coenzyme Q_10_ (CoQ_10_) is reduced to ubiquinol (CoQ_10_H_2_) by FSP1 at the plasma membrane and by DHODH at the mitochondrial inner membrane; ubiquinol traps lipid radicals and limits propagation of peroxidation. The GCH1–BH_4_ axis provides additional radical-trapping antioxidant capacity. When these defenses are overwhelmed, lipid peroxidation escalates and culminates in ferroptotic cancer cell death. *Abbreviations:* AA, arachidonic acid; AdA, adrenic acid; ACSL4, acyl-CoA synthetase long-chain family member 4; ALOX12/15, 12/15-lipoxygenase; BH_4_, tetrahydrobiopterin; CoQ_10_, coenzyme Q_10_ (ubiquinone); CoQ_10_H_2_, ubiquinol; DHODH, dihydroorotate dehydrogenase; Fe^2+^/Fe^3+^, ferrous/ferric iron; FSP1 (AIFM2), ferroptosis suppressor protein 1; GCH1, GTP cyclohydrolase-1; GPX4, glutathione peroxidase 4; GR, glutathione reductase; GSH, reduced glutathione; GSSG, oxidized glutathione; GTP, guanosine triphosphate; Glu, glutamate; Gly, glycine; IMM, inner mitochondrial membrane; ISC, iron-sulfur cluster; LPCAT3, lysophosphatidylcholine acyltransferase 3; NAD(P)H/NAD(P)^+^, reduced/oxidized nicotinamide adenine dinucleotide (phosphate); O_2_, molecular oxygen; PUFA-PL, polyunsaturated-fatty-acid phospholipid; PUFA-PL-OH, phospholipid alcohol; PUFA-PL-OO•, phospholipid peroxyl radical; PUFA-PL-OOH, phospholipid hydroperoxide; ROS, reactive oxygen species; Se, selenium; SLC3A2, heavy chain of system xc^−^; SLC7A11 (xCT), light chain of system xc^−^; system xc^−^, cystine/glutamate antiporter; TCA, tricarboxylic acid cycle.

Ferroptosis is a regulated form of cell death that is mechanistically distinct from apoptosis, necroptosis, and pyroptosis, and it is primarily driven by the iron-dependent accumulation of lipid peroxides within cellular membranes [1,2]. Since its discovery, ferroptosis has garnered substantial interest because of its close link with redox homeostasis, iron metabolism, and lipid biology, positioning it as a unique vulnerability in malignant cells characterized by metabolic reprogramming and therapy resistance [3,4]. The execution of ferroptosis involves uncontrolled oxidative modification of polyunsaturated fatty acids (PUFAs) in phospholipids, which disrupts membrane integrity and culminates in cell death [5]. This process is tightly regulated by multiple antioxidant defense systems, such as system x_c_^−^, glutathione peroxidase 4 (GPX4), ferroptosis suppressor protein 1 (FSP1), and, more recently, mitochondrial dihydroorotate dehydrogenase (DHODH), which was identified as an additional protective system that curtails mitochondrial lipid peroxidation (Figure 1) [6,7].

DHODH is a flavin-dependent enzyme embedded in the inner mitochondrial membrane that catalyzes the fourth step of de novo pyrimidine biosynthesis by oxidizing dihydroorotate to orotate, transferring electrons to coenzyme Q (ubiquinone), and linking nucleotide metabolism to mitochondrial respiration [8]. Beyond its canonical role in nucleotide synthesis, DHODH is increasingly recognized as a metabolic-signaling hub that regulates cancer cell proliferation, stress adaptation, and survival under hostile microenvironmental conditions [9]. Importantly, Mao and colleagues demonstrated in 2021 that DHODH serves as a mitochondria-specific ferroptosis defense system by reducing ubiquinone (CoQ_10_) to ubiquinol (CoQ_10_H_2_), thereby limiting the propagation of lipid peroxyl radicals and preserving mitochondrial membrane integrity [6]. This discovery has shifted DHODH from being regarded solely as a nucleotide metabolic enzyme to being recognized as a critical determinant of ferroptosis sensitivity, particularly in GPX4-low tumor contexts.

The intersection of DHODH with ferroptosis has profound implications for oncology. Cancer cells frequently develop resistance to conventional cytotoxic and targeted therapies, but this often comes at the cost of heightened dependency on ferroptosis defense mechanisms [10,11]. Tumors with diminished GPX4 expression or impaired cystine uptake become increasingly reliant on mitochondrial DHODH to suppress lipid peroxidation, rendering them selectively vulnerable to DHODH inhibition [7,12]. Consequently, DHODH inhibitors such as brequinar, leflunomide, and novel derivatives have been revisited as potential anticancer agents capable of inducing ferroptosis and overcoming drug resistance in malignancies such as glioblastoma, neuroblastoma, hepatocellular carcinoma, colorectal cancer, and triple-negative breast cancer [13,14,15,16,17].

Recent preclinical studies have provided compelling evidence that pharmacological targeting of DHODH synergizes with GPX4 inhibitors, immune checkpoint blockade, or standard chemotherapeutics to enhance ferroptotic cell death and restrict tumor progression [18,19]. For instance, in glioblastoma models resistant to temozolomide, DHODH inhibition disrupts mitochondrial antioxidant defenses, sensitizing tumors to ferroptosis and improving survival outcomes [20]. In hepatocellular carcinoma, low GPX4 expression amplifies susceptibility to DHODH blockade, leading to robust ferroptotic responses and enhanced therapeutic efficacy [21]. Furthermore, studies indicate that DHODH regulates not only tumor intrinsic metabolic survival programs but also shapes the tumor immune microenvironment by modulating antigen presentation, lipid remodeling, and T cell cytotoxicity [19,22]. These findings support the concept of DHODH as a dual-function target that bridges metabolic rewiring and immune evasion.

Parallel to these developments, nanomedicine and biomaterials approaches have introduced new possibilities for DHODH-targeted therapy. Rationally designed nanoparticles and nanozymes that co-deliver DHODH inhibitors alongside ferroptosis inducers or immunotherapeutics have demonstrated remarkable potency in preclinical cancer models by simultaneously disabling multiple ferroptosis defense arms [17,23]. These combinatorial strategies exploit the multifaceted role of DHODH in redox regulation and nucleotide metabolism, offering innovative avenues to circumvent therapy resistance.

Despite the growing excitement, challenges remain. Clinical translation of ferroptosis-targeted therapies has been limited by concerns over toxicity, off-target effects, and a lack of reliable biomarkers to stratify patients who would most benefit from DHODH inhibition [2,7]. Furthermore, the complex redundancy among ferroptosis defense systems and the metabolic plasticity of tumors complicate therapeutic exploitation of this pathway. A deeper understanding of context-specific dependencies, such as the interplay between DHODH and GPX4 levels, the tumor lipidome, and immune microenvironmental cues, will be critical for rational clinical application. In this review, we aim to synthesize recent progress at the intersection of DHODH, ferroptosis, and cancer biology. We first outline the biochemical structure and canonical role of DHODH in pyrimidine metabolism, followed by a discussion of its non-canonical function in mitochondrial ferroptosis defense. We then examine the diverse contributions of DHODH to tumor progression, therapy resistance, and immune evasion. Subsequently, we highlight therapeutic strategies targeting DHODH, including small-molecule inhibitors, nanomedicine approaches, and combinatorial regimens that exploit ferroptosis vulnerabilities. Finally, we provide perspectives on translational challenges, biomarker development, and future directions for integrating DHODH-based strategies into precision oncology.

## 2. Dihydroorotate Dehydrogenase: Structure, Function, and Metabolic Roles

**Figure 2:**
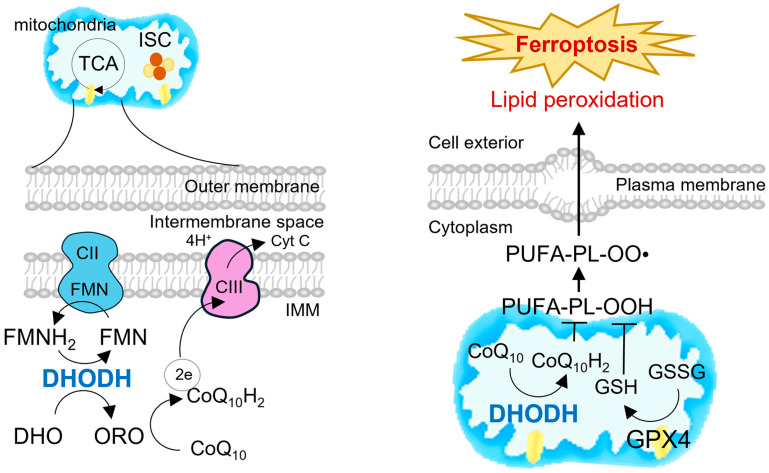
Coupling of de novo pyrimidine synthesis to the respiratory chain and mitochondria-specific lipid-peroxidation defense via DHODH. Electron-transfer topology at the inner mitochondrial membrane (**left column**). Membrane-anchored DHODH oxidizes dihydroorotate (DHO) to orotate (ORO) through its FMN cofactor (FMN/FMNH_2_), reduces coenzyme Q (CoQ_10_) to ubiquinol (CoQ_10_H_2_), and feeds electrons into complex III (CIII) to support proton translocation and cytochrome c (Cyt C) reduction; complex II (CII) is shown as an additional contributor to the ubiquinone pool, with TCA cycle and iron–sulfur cluster (ISC) biogenesis providing mitochondrial context. Mitochondria-confined ferroptosis defense (**right column**). DHODH continuously regenerates CoQ_10_H_2_, which traps phospholipid peroxyl radicals (PUFA-PL-OO•) and limits the propagation of lipid peroxidation. In parallel, mitochondrial GPX4 uses GSH (GSH→GSSG) to reduce phospholipid hydroperoxides (PUFA-PL-OOH) to PUFA-PL-OH, together restraining peroxidative spread toward the plasma membrane. Loss or inhibition of DHODH and/or GPX4 depletes CoQ_10_H_2_ and GSH pools, permitting unchecked lipid peroxidation and culminating in ferroptotic cell death. CII, complex II; CIII, complex III; Cyt C, cytochrome c; FMNH_2_, reduced flavin mononucleotide.

DHODH is a flavin-dependent oxidoreductase localized to the inner mitochondrial membrane, where it couples de novo pyrimidine biosynthesis with the mitochondrial respiratory chain [24,25]. As the only mitochondrial enzyme in this pathway, DHODH catalyzes the oxidation of dihydroorotate (DHO) to orotate (ORO), with electrons transferred to CoQ_10_ and further shuttled into complex III of the electron transport chain (Figure 2) [26,27]. This reaction not only supplies the precursors required for nucleotide synthesis but also directly contributes to redox homeostasis by maintaining the CoQ_10_/CoQ_10_H_2_ pool [28,29]. Consequently, DHODH has emerged as a key node at the interface of nucleotide metabolism, mitochondrial function, and regulated cell death pathways such as ferroptosis [9,30].

### 2.1. Structural Organization and Evolutionary Conservation

Human DHODH belongs to class II DHODHs, which are membrane-associated monomeric enzymes present in higher eukaryotes [31]. In contrast, prokaryotic and yeast orthologs (class I DHODHs) are cytosolic and rely on soluble electron acceptors such as fumarate or NAD^+^ rather than CoQ_10_ [32,33]. Class II enzymes contain an *N*-terminal mitochondrial targeting sequence and a hydrophobic domain that anchors them to the outer face of the inner mitochondrial membrane, allowing direct electron transfer to CoQ_10_ [34]. The catalytic domain harbors a flavin mononucleotide (FMN)-binding pocket essential for DHO oxidation, and structural studies have revealed conformational adaptations that facilitate substrate binding and electron transfer [35]. Despite limited sequence similarity across species, the preservation of this catalytic architecture underscores the evolutionary importance of DHODH in sustaining cellular proliferation and mitochondrial energy metabolism [28].

### 2.2. Canonical Role in Pyrimidine Metabolism

The de novo pyrimidine biosynthetic pathway is indispensable for rapidly proliferating cells, including malignant cells, because the salvage pathway alone cannot meet the heightened demand for nucleotides [36]. DHODH catalyzes the rate-limiting fourth step of this pathway, which follows the generation of carbamoyl aspartate by the multifunctional CAD complex. The oxidation of DHO to ORO by DHODH is coupled with CoQ_10_ reduction, linking pyrimidine biosynthesis to oxidative phosphorylation [37,38]. The downstream conversion of ORO to uridine monophosphate (UMP) by uridine monophosphate synthetase (UMPS) provides the central precursor for uridine triphosphate (UTP) and cytidine triphosphate (CTP), which are required for RNA synthesis, phospholipid production, and glycosylation reactions [39]. In addition, de novo pyrimidines feed into DNA synthesis via deoxyribonucleotide production, underscoring the pivotal role of DHODH in genome replication and repair [40,41].

In physiological settings, quiescent cells can often rely on salvage pathways, but proliferating lymphocytes, hematopoietic progenitors, and tumor cells depend heavily on de novo pyrimidine synthesis, rendering DHODH a metabolic bottleneck in these contexts [38]. This dependency has led to the development of DHODH inhibitors, originally for autoimmune diseases such as rheumatoid arthritis and multiple sclerosis (leflunomide and its active metabolite teriflunomide), and later repurposed for anticancer applications [42,43].

### 2.3. Integration with Mitochondrial Electron Transport and Redox Control

Beyond nucleotide biosynthesis, DHODH is intricately linked with mitochondrial redox homeostasis. By donating electrons to ubiquinone, DHODH contributes to the regeneration of CoQ_10_H_2_, a lipophilic antioxidant that scavenges lipid peroxyl radicals and suppresses mitochondrial lipid peroxidation [44]. This function positions DHODH alongside GPX4 and FSP1 as a parallel ferroptosis defense system, acting specifically within mitochondria [45]. When DHODH activity is impaired, the ubiquinol pool is depleted, promoting the accumulation of reactive oxygen species (ROS) and amplifying mitochondrial oxidative damage [46,47]. This dual role supporting both biosynthetic metabolism and antioxidant defense explains why DHODH inhibition exerts both cytostatic and cytotoxic effects in cancer cells [13].

Recent evidence further highlights that DHODH activity influences glycolysis and tricarboxylic acid (TCA) cycle fluxes through modulation of the NAD^+^/NADH ratio and mitochondrial membrane potential [8]. By coupling pyrimidine synthesis to energy production, DHODH functions as a metabolic integrator, allowing cancer cells to adapt to hypoxia, nutrient deprivation, or oxidative stress [19,48,49]. Thus, inhibition of DHODH simultaneously disrupts nucleotide availability, mitochondrial respiration, and ferroptosis defense, offering a multifaceted therapeutic strategy [14,50].

### 2.4. Non-Canonical Functions and Signaling Roles

In addition to its enzymatic role, DHODH exerts broader influences on oncogenic signaling networks. For example, DHODH interacts with c-Myc, stabilizing it independently of catalytic activity and thereby promoting tumorigenesis [51]. It also contributes to metabolic rewiring that favors epithelial–mesenchymal transition, immune evasion, and drug resistance [9,22]. Moreover, DHODH has been implicated in regulating macropinocytosis, supporting nutrient scavenging in nutrient-limited tumor microenvironments and indirectly shaping the immune landscape [22]. These findings highlight the dual enzymatic and non-enzymatic roles of DHODH, extending its relevance beyond pyrimidine metabolism to encompass signaling, adaptation, and survival pathways.

In addition to its catalytic role, emerging evidence reveals that DHODH exerts multiple non-enzymatic signaling functions that critically influence oncogenic pathways. Notably, DHODH directly interacts with and stabilizes c-Myc protein, thereby sustaining transcriptional programs involved in nucleotide biosynthesis, mitochondrial biogenesis, and ferroptosis resistance [51]. This stabilization occurs independently of its dehydrogenase activity and is mediated by protein–protein interactions within the mitochondrial outer membrane. Moreover, DHODH undergoes post-translational modifications such as ubiquitination and acetylation that regulate its stability and signaling potential under metabolic stress [9]. Beyond c-Myc, DHODH interfaces with STAT3 and β-catenin signaling to promote epithelial–mesenchymal transition and redox adaptation, emphasizing its dual role as a metabolic enzyme and signaling scaffold [22]. Collectively, these non-enzymatic functions link DHODH to key oncogenic and metabolic networks, expanding its biological relevance beyond pyrimidine metabolism.

### 2.5. Implications for Cancer Biology

Because of its unique localization and pleiotropic roles, DHODH is frequently upregulated in malignancies, including colorectal cancer, hepatocellular carcinoma, glioblastoma, breast cancer, and renal cell carcinoma [13,15,16,17]. Elevated DHODH expression correlates with enhanced proliferative capacity, metabolic plasticity, and poor prognosis [52,53,54]. Its function as a mitochondrial ferroptosis defense arm also implies that tumors with high oxidative stress or low GPX4 levels may exhibit selective vulnerability to DHODH blockade [6]. Thus, DHODH represents both a metabolic vulnerability and a signaling hub, making it an attractive therapeutic target across diverse cancers.

## 3. Mitochondrial Ferroptosis Defense: DHODH in Context

**Figure 3:**
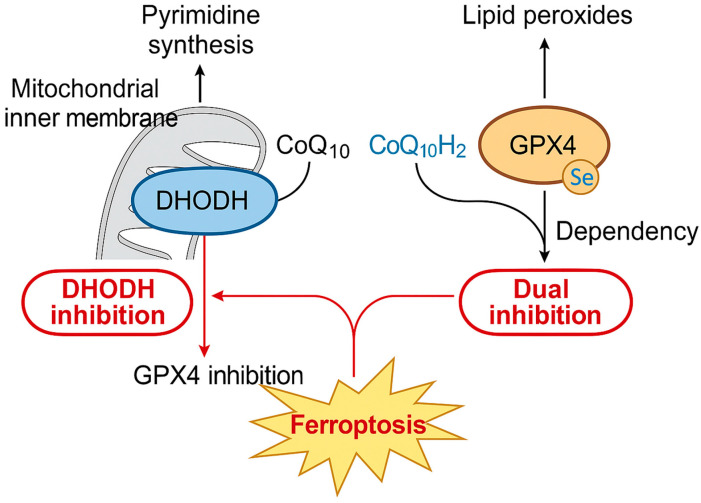
Crosstalk and compensatory mechanisms between DHODH and GPX4 pathways in ferroptosis. Schematic representation showing the cooperative antioxidant defenses mediated by DHODH and GPX4. DHODH, located in the mitochondrial inner membrane, supports pyrimidine synthesis and regenerates reduced coenzyme Q_10_ (CoQ_10_H_2_) to suppress mitochondrial lipid peroxidation. GPX4, a selenoenzyme in the cytosol and mitochondria, detoxifies lipid peroxides using glutathione (GSH). When GPX4 activity is inhibited, cells become increasingly dependent on DHODH to maintain redox balance. Dual inhibition of GPX4 and DHODH abolishes these compensatory defenses, leading to excessive lipid peroxide accumulation and synthetic lethal ferroptosis.

The regulation of ferroptosis is orchestrated by multiple antioxidant defense systems that act across distinct cellular compartments. Among these, system x_c_^−^, GPX4, FSP1, and DHODH represent the core enzymatic barriers preventing the unchecked accumulation of lipid peroxides [2,3]. While GPX4 is broadly recognized as the cytosolic and mitochondrial guardian against lipid hydroperoxides, and FSP1 functions at the plasma membrane to regenerate reduced ubiquinol, DHODH has more recently emerged as a mitochondria-specific ferroptosis defense arm that directly suppresses lipid peroxidation within the inner mitochondrial membrane [6,7]. As illustrated in Figure 3, inhibition of GPX4 enhances cellular dependence on DHODH for mitochondrial redox homeostasis, and combined inhibition of both pathways triggers synthetic lethal ferroptosis.

### 3.1. Parallel Antioxidant Systems in Ferroptosis Defense

GPX4 detoxifies phospholipid hydroperoxides by catalyzing their reduction to lipid alcohols using glutathione (GSH) as a cofactor, thereby curbing ferroptosis execution [55,56]. Its activity is tightly dependent on cysteine availability and system x_c_^−^ function, which maintains intracellular GSH pools [57,58]. In contrast, FSP1 mediates ferroptosis resistance by reducing ubiquinone to ubiquinol at the plasma membrane in an NAD(P)H-dependent manner, acting independently of GPX4 [59,60,61]. The discovery of DHODH added a new dimension to this regulatory network, as it operates within mitochondria to maintain redox balance specifically against lipid peroxidation at the site of high reactive oxygen species (ROS) generation [6,26].

Together, these three systems exemplify the principle of spatially segregated ferroptosis defense. GPX4 primarily safeguards the cytoplasm and mitochondrial matrix, FSP1 acts at the plasma membrane, and DHODH defends the mitochondrial inner membrane [7,9]. This compartmentalization allows cells to mount context-specific responses depending on the source of oxidative stress and the metabolic state of the cell.

### 3.2. DHODH as a Mitochondrial Ferroptosis Checkpoint

DHODH uniquely integrates nucleotide metabolism with mitochondrial antioxidant defense. By reducing ubiquinone to ubiquinol (CoQ_10_H_2_), DHODH replenishes the mitochondrial pool of lipophilic antioxidants, limiting lipid peroxyl radical propagation and mitochondrial membrane damage [6,62]. In tumors with diminished GPX4 expression, DHODH becomes the dominant ferroptosis defense, and its inhibition selectively sensitizes such cells to lipid peroxidation and ferroptotic death [7,42]. Conversely, cancers with high GPX4 expression display relative resistance to DHODH blockade, underscoring the interdependence of these parallel systems [13].

Recent studies have also shown that DHODH inhibition synergizes with ferroptosis inducers such as erastin or RSL3, amplifying lipid peroxidation and tumor cell death [14,15]. Supplementation with mitochondrial-targeted ubiquinol analogs, such as mitoQH_2_, can rescue DHODH-deficient cells, confirming the central role of DHODH-CoQ_10_H_2_ redox cycling in maintaining mitochondrial membrane integrity [7]. These findings position DHODH as a “mitochondrial checkpoint” that determines ferroptosis susceptibility, particularly under metabolic stress conditions.

### 3.3. Crosstalk Between DHODH and GPX4/FSP1 Pathways

Although DHODH operates independently, functional crosstalk exists among the three major ferroptosis defense systems. Inhibition of DHODH enhances the vulnerability of cells with low GPX4 activity, suggesting a compensatory relationship between these systems [2,6]. Similarly, combined blockade of DHODH and FSP1 synergistically promotes ferroptosis by disabling both mitochondrial and plasma membrane CoQ_10_ reduction mechanisms [18,63]. This redundancy illustrates the evolutionary importance of lipid peroxide detoxification and highlights potential therapeutic strategies to overcome cancer resistance by simultaneous targeting of multiple ferroptosis defenses [64].

Emerging evidence also suggests that DHODH interacts with other redox regulatory pathways, including the KEAP1-NRF2 axis and the mevalonate pathway. For example, DHODH inhibition perturbs cholesterol and lipid biosynthesis, which can amplify ferroptosis through altered phospholipid composition [15]. Additionally, tumors with KEAP1 mutations, which rely on NRF2-driven antioxidant programs, may exhibit synthetic vulnerabilities when DHODH is targeted in conjunction with GPX4 or FSP1 inhibition [19,65].

### 3.4. Implications for Mitochondrial Ferroptosis in Cancer

The identification of DHODH as a mitochondria-specific ferroptosis defense system has profound implications for cancer therapy. Many tumors exhibit metabolic heterogeneity, with subsets of cells displaying high oxidative stress or reduced GPX4 activity [66]. In these contexts, DHODH provides an essential buffer against mitochondrial lipid peroxidation, enabling tumor survival under adverse conditions [13,22,67]. Targeting DHODH can therefore selectively eliminate cancer cells that are otherwise refractory to apoptosis or necroptosis, while sparing normal cells that rely more heavily on salvage pathways and cytosolic GPX4 for redox protection [17,51].

Furthermore, mitochondrial ferroptosis regulation has been linked to therapy resistance. Glioblastomas and hepatocellular carcinomas with high metabolic stress adapt by upregulating DHODH, and inhibition of this enzyme restores sensitivity to chemotherapeutics such as temozolomide and platinum drugs [14,21,68]. In addition, DHODH-mediated ferroptosis defense has been shown to shape the tumor immune microenvironment by modulating lipid peroxidation, antigen presentation, and T cell infiltration [19,22]. These findings suggest that DHODH functions as both a metabolic and immunological gatekeeper, influencing not only cell-autonomous survival but also tumor-immune interactions.

## 4. DHODH and Cancer Progression

The link between DHODH and cancer has gained considerable attention in recent years as multiple studies have demonstrated its involvement not only in nucleotide metabolism but also in redox homeostasis, signaling adaptation, and ferroptosis regulation [7,9]. Unlike normal differentiated cells, which can rely on salvage pathways for nucleotide supply, proliferating tumor cells depend heavily on de novo pyrimidine biosynthesis, making DHODH a metabolic bottleneck that sustains uncontrolled cell growth [69,70]. Moreover, DHODH expression is frequently upregulated in a wide range of malignancies and is strongly correlated with tumor aggressiveness, metastasis, and poor prognosis [71,72,73] (Table 1).

### 4.1. DHODH Expression Across Cancer Types

Transcriptomic and proteomic analyses have consistently revealed that DHODH is highly expressed in rapidly proliferating tumors, including hepatocellular carcinoma, colorectal cancer, glioblastoma, neuroblastoma, breast cancer, melanoma, and renal cell carcinoma [13,14,15,16,17]. Elevated DHODH levels have been associated with worse clinical outcomes, including reduced overall survival and resistance to therapy [14,20]. For example, in colorectal cancer, redistribution of DHODH from mitochondria to the cytosol was shown to drive 5-fluorouracil (5-FU) resistance by sustaining pyrimidine synthesis independently of mitochondrial metabolism, thereby dampening drug efficacy [13]. In glioblastoma, DHODH stabilization by PRR11 was found to maintain mitochondrial function and ferroptosis resistance, further enhancing temozolomide resistance [14]. Similarly, in neuroblastoma, inhibition of DHODH reprograms lipid metabolism through the mevalonate pathway, thereby promoting ferroptosis sensitivity and revealing a novel therapeutic vulnerability [15].

### 4.2. Oncogenic Signaling and Metabolic Reprogramming

Beyond its canonical metabolic role, DHODH also acts as a signaling hub in cancer. It interacts with oncogenes such as c-Myc, forming a feedback loop in which DHODH stabilizes Myc protein, while Myc transcriptionally upregulates DHODH expression [51]. This metabolic-signaling axis drives uncontrolled pyrimidine production, ferroptosis defense, and tumor progression, particularly in colorectal and hematologic malignancies. In breast cancer, the deubiquitinase USP24 stabilizes DHODH protein, protecting tumor cells from ferroptosis by preventing DHODH degradation [16]. In hepatocellular carcinoma, the YBX1–RNF115 regulatory circuit promotes DHODH stability through ubiquitination control, further suppressing ferroptosis and supporting tumor growth [74]. These findings highlight how DHODH is embedded within complex post-translational and transcriptional networks that couple metabolic demands with oncogenic signaling.

### 4.3. DHODH and Therapy Resistance

Resistance to chemotherapy and radiotherapy remains a central challenge in oncology. Accumulating evidence suggests that DHODH plays a pivotal role in resistance mechanisms by maintaining nucleotide pools, stabilizing redox balance, and supporting DNA repair [8]. In glioblastoma, PRR11–DHODH interactions contribute to temozolomide resistance, while DHODH inhibition sensitizes tumors to ferroptosis and restores drug response [14]. In gastric and colorectal cancers, DHODH upregulation confers resistance to 5-FU and platinum-based chemotherapy by enhancing glycolytic flux and pyrimidine synthesis [13,21,68]. Importantly, pharmacological inhibition of DHODH with agents such as brequinar or leflunomide resensitizes resistant cells to chemotherapy, suggesting that targeting DHODH could serve as a strategy to overcome resistance in multiple tumor types [17,72,75,76].

Moreover, DHODH inhibition has been shown to impair DNA replication fork stability in PTEN-deficient cells, leading to replication stress and cell death [77]. In melanoma and skin cancers, DHODH supports UV-induced tumorigenesis through STAT3-mediated transcriptional activation, and its inhibition suppresses both primary tumor growth and metastatic progression [78]. Together, these findings establish DHODH as a central player in therapy resistance, with potential applications as both a predictive biomarker and therapeutic target.

### 4.4. Contribution to Immune Evasion and Tumor Microenvironment

Recent studies have expanded the role of DHODH beyond intrinsic tumor metabolism to include modulation of the tumor microenvironment and immune evasion. DHODH-driven macropinocytosis sustains nutrient acquisition under conditions of scarcity and simultaneously represses MHC class II expression, thereby reducing tumor immunogenicity and contributing to immune escape [22]. Inactivation of DHODH enhances T cell infiltration and potentiates the efficacy of PD-1 checkpoint blockade in mouse tumor models [19]. These findings suggest that DHODH may act as a metabolic checkpoint that integrates ferroptosis regulation with tumor immune evasion. Furthermore, the balance of mitochondrial lipid peroxidation regulated by DHODH influences the release of damage-associated molecular patterns (DAMPs), thereby shaping dendritic cell activation and anti-tumor immune responses [76]. The ability of DHODH to link mitochondrial metabolism with immune regulation positions it as a promising therapeutic target for combination strategies involving immunotherapy and ferroptosis induction [18].

### 4.5. Prognostic and Translational Significance

Given its roles in proliferation, resistance, and immune evasion, DHODH expression is increasingly recognized as a prognostic marker across cancer types. High DHODH levels correlate with poor clinical outcomes in colorectal cancer, hepatocellular carcinoma, glioblastoma, and triple-negative breast cancer [13,14,15,16,17]. The context-specific dependency of tumors on DHODH, particularly in GPX4-low or metabolically stressed environments, suggests that biomarker-driven patient stratification could maximize therapeutic benefit [7]. As clinical trials with DHODH inhibitors expand, identifying predictive markers of response, such as GPX4 expression, Myc status, or DHODH post-translational regulation, will be essential for precision oncology applications.

## 5. Therapeutic Targeting of DHODH in Ferroptosis-Induced Cancer Therapy

The identification of DHODH as a mitochondria-specific ferroptosis defense mechanism has positioned it as an attractive therapeutic target across a broad spectrum of malignancies [6,79]. Pharmacological inhibition of DHODH simultaneously disrupts de novo pyrimidine biosynthesis and mitochondrial redox homeostasis, generating a dual vulnerability that can be exploited for cancer therapy [42,50]. Importantly, cancer cells with diminished GPX4 expression or high oxidative stress are particularly dependent on DHODH activity, making them selectively sensitive to its inhibition [13,15,75]. This section summarizes the current therapeutic strategies targeting DHODH, ranging from classical inhibitors to emerging nanomedicine and combination approaches (Table 2).

### 5.1. Conventional DHODH Inhibitors and Repurposing Strategies

Several small-molecule DHODH inhibitors have been developed over the past decades, originally for autoimmune and infectious diseases, but many are now being evaluated in oncology. Leflunomide and its active metabolite, teriflunomide, which inhibit DHODH activity, have long been used in the treatment of rheumatoid arthritis and multiple sclerosis [79,80,81]. Brequinar, a potent and selective inhibitor, was initially investigated as an anticancer drug in the 1990s but was abandoned due to dose-limiting toxicities and limited clinical responses [19,46,82]. With the renewed recognition of DHODH’s role in ferroptosis, brequinar or its liposome has resurfaced as a promising candidate for combination therapy in resistant tumors such as glioblastoma, colorectal cancer, and hepatocellular carcinoma [13,14,74,76]. Recent efforts have also identified novel DHODH inhibitors with improved selectivity and pharmacokinetics. Piperine derivatives, caffeic acid phenethyl ester (CAPE), and other plant-derived molecules have demonstrated preclinical efficacy in promoting ferroptosis through DHODH inhibition [16,83]. A novel orally bioavailable DHODH inhibitor, BAY 2402234, showed monotherapy efficacy in the treatment of myeloid leukemias [84]. Human DHODH inhibitors have been developed with the context of maximum tumoricidal and minimal adverse effects [81]. These compounds often synergize with ferroptosis inducers, highlighting their translational potential.

### 5.2. Combination Therapy Strategies

Monotherapy with DHODH inhibitors often results in adaptive resistance, largely due to compensatory metabolic pathways such as pyrimidine salvage or FSP1-mediated redox defense [7]. Consequently, combination strategies are being explored to maximize therapeutic efficacy. One approach involves co-targeting the GSH-GPX4 axis and DHODH. Simultaneous inhibition of these two defense arms synergistically enhances lipid peroxidation and ferroptotic cell death, even in resistant cancers [45,85,86,87]. Similarly, dual inhibition of DHODH and FSP1 has shown promising results in colorectal and breast cancer models, as both enzymes converge on ubiquinol regeneration pathways [6,88].

Chemotherapy combinations are another avenue of interest. For instance, brequinar or leflunomide enhances the efficacy of temozolomide in glioblastoma, oxaliplatin in hepatocellular carcinoma, and 5-fluorouracil in gastric and colorectal cancer [13,14,80]. Targeting DHODH also sensitizes tumors to radiotherapy by amplifying oxidative damage and ferroptosis [20,75]. Importantly, in PTEN- or LKB1-deficient tumors, DHODH inhibition exploits synthetic vulnerabilities linked to replication stress and defective nucleotide metabolism [37,77,89]. Immunotherapy combinations are emerging as particularly promising. DHODH inhibition promotes ferroptosis-mediated release of DAMPs, enhances dendritic cell maturation, and boosts CD8^+^ T cell cytotoxicity [22,76]. Furthermore, pharmacological targeting of DHODH reverses PD-1 resistance by disrupting tumor macropinocytosis and restoring antigen presentation [22]. These findings underscore DHODH inhibition as a potent adjuvant to immune checkpoint blockade.

### 5.3. Nanomedicine and Targeted Delivery Platforms

Given the systemic toxicities associated with conventional inhibitors, nanotechnology-based delivery systems have been developed to enhance tumor specificity and reduce adverse effects. Recent studies have engineered multifunctional nanoparticles that co-deliver DHODH inhibitors with ferroptosis inducers, iron donors, or immunotherapeutic agents [17,23]. For example, calcium phosphate-mineralized nanoplatforms incorporating brequinar, erastin, and iFSP1 demonstrated robust ferroptosis induction in triple-negative breast cancer while simultaneously enhancing anti-PD-L1 therapy efficacy [18]. Similarly, metal–phenolic network nanoparticles carrying DHODH inhibitors and temozolomide were shown to overcome drug resistance in glioblastoma by disrupting multiple ferroptosis defense arms [20]. In pancreatic cancer, mitocytosis-inducing nanoparticles co-delivering gemcitabine and leflunomide alleviated chemotherapy resistance by simultaneously disrupting pyrimidine synthesis and redox homeostasis [17]. In breast cancers, nanoparticles carrying DHODH inhibitors enhanced lipid peroxidation and ferroptosis with no significant adverse effects [90]. These approaches highlight the potential of rationally designed nanoplatforms to dismantle the multi-layered ferroptosis resistance networks in tumors.

### 5.4. Challenges and Translational Considerations

Despite preclinical success, several challenges remain for DHODH-targeted therapies. First, systemic inhibition of DHODH may affect proliferating normal cells such as lymphocytes and hematopoietic progenitors, raising concerns about toxicity [9]. Second, metabolic plasticity in tumors, including upregulation of pyrimidine salvage pathways, can attenuate the efficacy of DHODH inhibitors [13]. Third, reliable biomarkers to identify patients most likely to benefit from DHODH inhibition are still lacking. Expression of DHODH, GPX4, and FSP1, as well as specific oncogenic alterations such as Myc amplification, may serve as predictive indicators but require clinical validation [15,51]. Additionally, the dual enzymatic and non-enzymatic roles of DHODH complicate therapeutic targeting [51]. Inhibitors that block its catalytic activity may not fully disrupt its oncogenic protein–protein interactions, suggesting that dual-function inhibitors or combination regimens may be necessary for durable responses [50,91].

### 5.5. Clinical Perspectives

The therapeutic exploitation of DHODH in ferroptosis-induced cancer therapy is at an exciting juncture. As the understanding of DHODH expands from pyrimidine metabolism to redox control and immune modulation, targeting this enzyme offers a unique opportunity to simultaneously disrupt cancer cell proliferation, ferroptosis resistance, and immune evasion [7,19]. Rational drug design, integration of nanomedicine, and biomarker-driven patient selection will be critical to advancing DHODH inhibitors into clinical practice. Moreover, combinatorial strategies with immunotherapy, radiotherapy, and chemotherapy hold the potential to overcome the multifactorial resistance mechanisms that limit current cancer treatments [9,92].

## 6. DHODH, Ferroptosis, and Tumor Immunity

The immune system plays a decisive role in determining the success or failure of anticancer therapies. Accumulating evidence shows that ferroptosis, beyond being a metabolic vulnerability, has profound effects on tumor-immune interactions [2,3]. The mitochondria-localized enzyme DHODH has emerged as a central regulator in this context, influencing both cancer cell-intrinsic ferroptosis defenses and the composition of the tumor immune microenvironment [19,22].

### 6.1. Ferroptosis and Immunogenic Cell Death

Ferroptosis has been increasingly recognized as a form of immunogenic cell death (ICD), capable of releasing DAMPs, lipid peroxidation products, and oxidized phospholipids that recruit and activate dendritic cells (DCs) and cytotoxic T lymphocytes (CTLs) [4,93]. The extent of ferroptosis within tumors strongly influences antigen presentation and T cell infiltration. By suppressing mitochondrial lipid peroxidation, DHODH can dampen ferroptosis-associated immunogenic signals, thereby limiting antitumor immunity [7]. Conversely, DHODH inhibition amplifies ferroptosis and enhances immune recognition of tumor cells, supporting its potential use as an immunotherapy adjuvant [19].

### 6.2. DHODH-Mediated Metabolic Adaptation and Immune Evasion

One mechanism by which tumors evade immune attack involves metabolic rewiring of pyrimidine biosynthesis and redox pathways. Recent work has shown that DHODH sustains tumor macropinocytosis through O-GlcNAcylation of neuropilin-1 (NRP1), which promotes nutrient scavenging and suppresses MHC class II expression on tumor cells [22]. This process reduces antigen visibility and blunts T cell responses. Pharmacological inhibition of DHODH restored MHC-II expression, increased CD8^+^ T cell infiltration, and reversed resistance to PD-1 blockade in preclinical models [22]. Similarly, DHODH-driven regulation of the CDP–choline pathway affects phosphatidylcholine metabolism and lipid remodeling, influencing the abundance of immunosuppressive lipid species that hinder T cell activity [19].

### 6.3. Crosstalk with Immune Checkpoint Therapy

Immune checkpoint inhibitors (ICIs), including PD-1/PD-L1 and CTLA-4 blockade, have transformed cancer therapy but remain limited by primary and acquired resistance [94,95]. Ferroptosis induction has been shown to synergize with ICIs by increasing tumor immunogenicity and overcoming immunosuppressive barriers [96,97,98]. In this context, DHODH inhibition acts as a sensitizer that promotes ferroptosis, enhances T cell infiltration, and facilitates ICI efficacy. For example, in breast and lung cancer models, dual targeting of DHODH and PD-1/PD-L1 pathways significantly improved tumor control compared to either therapy alone [18,19]. These findings highlight DHODH as a metabolic checkpoint that can be leveraged to amplify immunotherapy responses.

### 6.4. Tumor Microenvironment Reprogramming by DHODH Inhibition

The tumor microenvironment (TME) is characterized by oxidative stress, hypoxia, and nutrient competition, conditions that shape both cancer progression and immune activity [99,100]. By maintaining mitochondrial ubiquinol pools, DHODH reduces lipid peroxidation and helps tumors adapt to oxidative pressure, thereby creating an immunosuppressive niche [7]. Inhibition of DHODH disrupts this balance, leading to accumulation of ROS and lipid peroxides that promote immunogenic ferroptosis and T cell activation [46]. Additionally, DHODH blockade has been linked to increased infiltration of interferon-γ–secreting CD8^+^ T cells, further enhancing antitumor immunity [19,101].

Nanoparticle-based delivery of DHODH inhibitors has also been shown to remodel the TME. For instance, multifunctional nanoplatforms that simultaneously inhibit DHODH, GPX4, and FSP1 not only enhance ferroptosis but also trigger maturation of DCs and recruitment of CTLs, thereby amplifying immune checkpoint therapy [17,18]. Such strategies highlight the potential of integrating metabolic targeting with immune modulation to dismantle tumor defenses.

### 6.5. Translational and Clinical Perspectives

From a translational perspective, DHODH inhibition represents a dual-action strategy: directly sensitizing tumor cells to ferroptosis and indirectly enhancing immune surveillance. Patient stratification based on DHODH expression, GPX4 levels, and immune gene signatures may enable precision deployment of DHODH inhibitors in combination with immunotherapy [82]. Moreover, monitoring ferroptosis biomarkers such as lipid peroxidation products and CoQ_10_H_2_ levels could help assess treatment response in clinical settings [11]. Clinical trials investigating DHODH inhibitors in cancer are still in early phases, but the preclinical evidence suggests strong potential for synergy with ICIs and radiotherapy [18,76,82]. The integration of nanomedicine approaches may further reduce systemic toxicity and improve delivery specificity, paving the way for clinical translation [23].

In summary, DHODH is more than a mitochondrial enzyme in pyrimidine synthesis–it is a metabolic checkpoint that links ferroptosis regulation with tumor immunity. By sustaining antioxidant defenses and suppressing immunogenic lipid peroxidation, DHODH supports immune evasion. Conversely, its inhibition not only drives ferroptosis but also amplifies the efficacy of immune checkpoint therapies, offering a promising strategy to remodel the tumor immune microenvironment.

## 7. Conclusions and Perspectives

The discovery of ferroptosis has reshaped our understanding of regulated cell death and revealed metabolic vulnerabilities that can be exploited for cancer therapy. Within this context, DHODH has emerged as a pivotal player, not only as a canonical enzyme in de novo pyrimidine biosynthesis but also as a mitochondrial checkpoint that safeguards against lipid peroxidation [6,7]. The identification of DHODH as a ferroptosis regulator has established a direct mechanistic link between nucleotide metabolism, redox control, and tumor immunity, positioning this enzyme at the crossroads of cancer cell survival and therapeutic resistance.

The evidence accumulated to date suggests that DHODH fulfills dual and context-dependent roles in cancer biology. On the one hand, it maintains the pyrimidine pools required for rapid proliferation and genome replication [9]. On the other hand, it sustains mitochondrial redox homeostasis by regenerating ubiquinol, thereby shielding tumor cells from ferroptotic death [13,15]. These functions render DHODH indispensable for tumor progression, particularly under conditions of metabolic stress, hypoxia, or oxidative burden. Elevated DHODH expression has been reported in colorectal, hepatocellular, breast, renal, and brain cancers, where it correlates with poor prognosis, drug resistance, and immune evasion.

Therapeutically, DHODH inhibition offers a multifaceted strategy. Classical inhibitors such as brequinar and leflunomide, along with novel derivatives and natural product-based compounds, have demonstrated potent antitumor activity by inducing ferroptosis and suppressing proliferation. Combination strategies are especially promising, as dual inhibition of DHODH and GPX4 or FSP1 amplifies ferroptosis, while pairing DHODH inhibitors with chemotherapy, radiotherapy, or immune checkpoint blockade restores sensitivity and enhances clinical efficacy. Advances in nanomedicine further expand the therapeutic potential by enabling targeted delivery and reducing systemic toxicity. Nonetheless, several challenges remain before DHODH-targeted therapies can be broadly implemented. First, systemic inhibition of DHODH may impact normal proliferative tissues, raising concerns about hematological and immune-related toxicities. Second, tumor cells may escape via compensatory salvage pathways or redundant ferroptosis defenses, necessitating rational combination approaches. Third, predictive biomarkers to identify patients most likely to benefit from DHODH inhibition are still under development. Integration of molecular signatures such as GPX4 expression, Myc amplification, and DHODH post-translational regulation may enable biomarker-guided therapy.

In addition to DHODH, GPX4, and FSP1, several regulators of iron and lipid metabolism critically influence ferroptosis sensitivity. Iron metabolism determines the labile iron pool that fuels lipid peroxidation, whereas lipid metabolism shapes the membrane composition that defines oxidative vulnerability. Within the lipid metabolic pathway, stearoyl-CoA desaturase 1 (SCD1) generates monounsaturated fatty acids (MUFAs) that replace PUFAs in phospholipids, thereby conferring ferroptosis resistance. Fatty acid–binding proteins (FABPs) and lipid droplet–associated proteins also buffer oxidizable lipids and prevent excessive peroxidation. The interplay among these lipid metabolic regulators, DHODH, and GPX4 constitutes a multilayered defense network that fine-tunes ferroptotic susceptibility in cancer cells. Recognizing these interconnected metabolic pathways may facilitate novel therapeutic strategies to overcome ferroptosis resistance and improve treatment efficacy.

Early-generation DHODH inhibitors, including brequinar and leflunomide, exhibited promising preclinical efficacy but were limited by systemic toxicity in clinical trials. These toxicities stemmed largely from the inhibition of de novo pyrimidine synthesis in rapidly dividing normal cells such as hematopoietic progenitors, gastrointestinal epithelium, and immune lymphocytes. Dose-limiting side effects included myelosuppression, hepatotoxicity, and gastrointestinal disturbances. Moreover, brequinar displayed a narrow therapeutic window and poor pharmacokinetic properties, contributing to insufficient target exposure and off-target mitochondrial effects. These early limitations prompted the development of improved DHODH inhibitors and targeted nanodelivery systems to enhance tumor selectivity and minimize systemic toxicity [9,19,46,80].

Resistance to DHODH inhibition represents another critical challenge in translating ferroptosis-based therapy. Tumor cells may bypass DHODH blockade through activation of pyrimidine salvage pathways, enhanced expression of nucleoside transporters, or adaptive metabolic reprogramming that reduces dependence on de novo pyrimidine synthesis. In addition, compensatory activation of parallel antioxidant systems such as GPX4, FSP1, or GCH1–BH4 can restore redox balance and suppress ferroptosis. Upregulation or post-translational stabilization of DHODH itself has been observed in resistant tumor subclones [51,74]. Therefore, rational combination strategies—such as co-targeting DHODH with GPX4, FSP1, or DNA repair inhibitors—may be necessary to overcome resistance and sustain therapeutic efficacy [13,15,76].

Looking forward, several research directions are particularly compelling. Elucidating the non-canonical roles of DHODH in oncogenic signaling, immune modulation, and metabolic crosstalk will expand its therapeutic scope. Exploring DHODH inhibition in synergy with ferroptosis inducers, immunotherapies, and radiotherapy may unlock novel avenues to overcome resistance. The application of nanotechnology and biomaterials for tumor-specific delivery of DHODH inhibitors represents another promising frontier. Finally, rigorous clinical trials, coupled with biomarker development, are required to translate preclinical advances into effective and safe treatments for patients.

In conclusion, DHODH represents a unique metabolic and redox checkpoint that integrates pyrimidine synthesis, ferroptosis regulation, and immune evasion. Targeting this enzyme offers a powerful therapeutic strategy to dismantle cancer resilience and reprogram the tumor microenvironment. As research continues to unravel the complexity of DHODH biology, it holds the promise of transforming ferroptosis-based therapy from preclinical innovation to clinical reality.

**Table 1:** DHODH expression, functions, and cancer progression.

Cancer Type	DHODH Expression/Function	Mechanism	Clinical/Prognostic Implications	Therapeutic Response to DHODH Inhibition	Reference
Colorectal cancer (CRC)	Upregulated; redistribution in 5-FU resistance	Maintains pyrimidine pools independently of mitochondria	Promotes chemoresistance; poor prognosis	Brequinar or leflunomide resensitize 5-FU–resistant CRC cells to chemotherapy and induce ferroptosis	[13]
Glioblastoma (GBM)	Stabilized by PRR11	Prevents ferroptosis, enhances DNA repair	Confers temozolomide resistance	Brequinar restores temozolomide sensitivity by triggering mitochondrial ferroptosis	[14]
Neuroblastoma	High DHODH dependence	Rewires mevalonate/lipid metabolism	Targetable metabolic vulnerability; ferroptosis induction	DHODH blockade (brequinar) induces ferroptosis via the mevalonate pathway and suppresses tumor growth	[15]
Hepatocellular carcinoma (HCC)	DHODH stabilized by RNF115 via YBX1–m5C modification axis	RNF115 ubiquitinates DHODH (K27) to prevent autophagic degradation, suppressing ferroptosis	YBX1/RNF115–DHODH axis promotes HCC progression; high expression predicts poor survival	Leflunomide or brequinar enhance ferroptosis and improve oxaliplatin efficacy in HCC	[74]
Triple-negative breast cancer (TNBC)	Stabilized by USP24	Prevents DHODH degradation, suppresses lipid peroxidation	Ferroptosis resistance; poor prognosis	DHODH inhibition synergizes with ferroptosis inducers and immune checkpoint therapy	[16]
Skin cancers (UV-induced cSCC)	UVB-induced DHODH upregulation via STAT3	Drives pyrimidine synthesis reprogramming under UV stress	DHODH inhibition (leflunomide) blocks tumor initiation and enhances chemoprevention/combination therapy	Leflunomide suppresses UV-induced tumorigenesis and enhances chemopreventive efficacy	[78]

**Table 2:** Therapeutic strategies targeting DHODH in ferroptosis-related cancer therapy.

Therapeutic Approach	Agents/Platforms	Mechanism of Action	Synergy/Applications	Reference
Conventional inhibitors	Leflunomide, teriflunomide, brequinar	Block pyrimidine biosynthesis and CoQ reduction	Cytostatic + ferroptosis induction; effective in CRC, HCC, GBM	[46,80,81,82]
Novel small molecules	Piperine derivatives, CAPE	Direct DHODH inhibition, ferroptosis activation	Synergy with GPX4 inhibitors; potential low-toxicity options	[16,83]
Combination with chemotherapy	Brequinar + temozolomide (GBM), leflunomide + Oxaliplatin (HCC), brequinar + 5-FU (CRC)	Dual disruption of DNA synthesis and redox defense	Overcomes chemoresistance, enhances apoptosis + ferroptosis	[13,14,80]
Combination with ferroptosis inducers	Erastin, RSL3 with brequinar	Inhibit SLC7A11 or GPX4 + DHODH	Potent ferroptosis amplification; effective in GPX4-low tumors	[85,86,87]
Combination with FSP1 inhibition	Brequinar (high dose), DHODH inhibitors + iFSP1 or genetic FSP1 loss (CRC, breast cancer)	Dual blockade of ubiquinol regeneration (DHODH in mitochondria, FSP1 at plasma membrane)	Synergistic ferroptosis induction; context-specific vulnerability	[6,88]
Combination with immunotherapy	DHODH inhibitors + anti–PD-1/PD-L1	Increase lipid peroxidation and antigenicity; restore MHC-II	Reverse immune evasion, enhance T cell infiltration	[22,76]
Nanoplatforms	brequinar–erastin–iFSP1 nanoparticles; gemcitabine–leflunomide nanocomplex; metal–phenolic networks	Co-delivery of DHODH inhibitors with ferroptosis inducers/chemo drugs	Deep tumor penetration, reduced systemic toxicity, synergy with ICIs	[17,18,20]

## Data Availability

No new data were created or analyzed in this study.

## References

[1] Dixon S.J., Lemberg K.M., Lamprecht M.R., Skouta R., Zaitsev E.M., Gleason C.E., Patel D.N., Bauer A.J., Cantley A.M., Yang W.S. (2012). Ferroptosis: An iron-dependent form of nonapoptotic cell death. Cell.

[2] Ubellacker J.M., Dixon S.J. (2025). Prospects for ferroptosis therapies in cancer. Nat. Cancer.

[3] Stockwell B.R., Friedmann Angeli J.P., Bayir H., Bush A.I., Conrad M., Dixon S.J., Fulda S., Gascón S., Hatzios S.K., Kagan V.E. (2017). Ferroptosis: A Regulated Cell Death Nexus Linking Metabolism, Redox Biology, and Disease. Cell.

[4] Friedmann Angeli J.P., Krysko D.V., Conrad M. (2019). Ferroptosis at the crossroads of cancer-acquired drug resistance and immune evasion. Nat. Rev. Cancer.

[5] Chen F., Kang R., Tang D., Liu J. (2024). Ferroptosis: Principles and significance in health and disease. J. Hematol. Oncol..

[6] Mao C., Liu X., Zhang Y., Lei G., Yan Y., Lee H., Koppula P., Wu S., Zhuang L., Fang B. (2021). DHODH-mediated ferroptosis defence is a targetable vulnerability in cancer. Nature.

[7] Cao J., Chen X., Chen L., Lu Y., Wu Y., Deng A., Pan F., Huang H., Liu Y., Li Y. (2025). DHODH-mediated mitochondrial redox homeostasis: A novel ferroptosis regulator and promising therapeutic target. Redox Biol..

[8] Boukalova S., Hubackova S., Milosevic M., Ezrova Z., Neuzil J., Rohlena J. (2020). Dihydroorotate dehydrogenase in oxidative phosphorylation and cancer. Biochim. Biophys. Acta Mol. Basis Dis..

[9] Lin F., Li J., Zhou L., Yi R., Chen Y., He S. (2025). Targeting vulnerability in tumor therapy: Dihydroorotate dehydrogenase. Life Sci..

[10] Lei G., Zhuang L., Gan B. (2022). Targeting ferroptosis as a vulnerability in cancer. Nat. Rev. Cancer.

[11] Zhou Q., Meng Y., Li D., Yao L., Le J., Liu Y., Sun Y., Zeng F., Chen X., Deng G. (2024). Ferroptosis in cancer: From molecular mechanisms to therapeutic strategies. Signal Transduct. Target. Ther..

[12] Liu Y., Lu S., Wu L.L., Yang L., Yang L., Wang J. (2023). The diversified role of mitochondria in ferroptosis in cancer. Cell Death Dis..

[13] Dong S., Zhang M., Cheng Z., Zhang X., Liang W., Li S., Li L., Xu Q., Song S., Liu Z. (2024). Redistribution of defective mitochondria-mediated dihydroorotate dehydrogenase imparts 5-fluorouracil resistance in colorectal cancer. Redox Biol..

[14] Miao Z., Xu L., Gu W., Ren Y., Li R., Zhang S., Chen C., Wang H., Ji J., Chen J. (2024). A targetable PRR11-DHODH axis drives ferroptosis- and temozolomide-resistance in glioblastoma. Redox Biol..

[15] Shir J.C., Chen P.Y., Kuo C.H., Hsieh C.H., Chang H.Y., Lee H.C., Huang C.H., Hsu C.H., Hsu W.M., Huang H.C. (2025). DHODH Blockade Induces Ferroptosis in Neuroblastoma by Modulating the Mevalonate Pathway. Mol. Cell Proteom..

[16] Yang L., An X., Yang S., Lin X., Chen Z., Xue Q., Chen X., Wang Y., Yan D., Chen S. (2025). The deubiquitinase USP24 suppresses ferroptosis in triple-negative breast cancer by stabilizing DHODH protein. Cell Death Dis..

[17] Wang Y., Fan H., Chen Q., Song H., Li X., Su B., Sun T., Cheng L., Jiang C. (2025). Mitocytosis-inducing nanoparticles alleviate gemcitabine resistance via dual disruption of pyrimidine synthesis and redox homeostasis in pancreatic ductal adenocarcinoma. Biomaterials.

[18] Zhu X., Xie L., Liao Q., Tian J., Peng J., Chen Z., Song E., Song Y. (2025). Calcium phosphate-mineralized nanoplatform for enhanced ferroptosis and synergistic anti-PDL1 therapy in triple-negative breast cancer through multi-pathway targeting. Acta Biomater..

[19] Teng D., Swanson K.D., Wang R., Zhuang A., Wu H., Niu Z., Cai L., Avritt F.R., Gu L., Asara J.M. (2025). DHODH modulates immune evasion of cancer cells via CDP-Choline dependent regulation of phospholipid metabolism and ferroptosis. Nat. Commun..

[20] Yin N., Wang B., Wang Y., Tian L., Han S., Zheng B., Feng F., Song S., Zhang H. (2025). A Metal-Phenolic Network Nanoresensitizer Overcoming Glioblastoma Drug Resistance through the Metabolic Adaptive Strategy and Targeting Drug-Tolerant Cells. Nano Lett..

[21] Fan R., Deng A., Lin R., Zhang S., Cheng C., Zhuang J., Hai Y., Zhao M., Yang L., Wei G. (2024). A platinum(IV)-artesunate complex triggers ferroptosis by boosting cytoplasmic and mitochondrial lipid peroxidation to enhance tumor immunotherapy. MedComm.

[22] Wang Y., Chai Y., Liu Y., Liu X., Zhang Y., Yang Z., Yin S., Luo J., Li Z., Gu Y. (2025). Inhibition of tumor cell macropinocytosis driver DHODH reverses immunosuppression and overcomes anti-PD1 resistance. Immunity.

[23] Xue P., Zhuang H., Shao S., Bai T., Zeng X., Yan S. (2024). Engineering Biodegradable Hollow Silica/Iron Composite Nanozymes for Breast Tumor Treatment through Activation of the “Ferroptosis Storm”. ACS Nano.

[24] Fang J., Uchiumi T., Yagi M., Matsumoto S., Amamoto R., Takazaki S., Yamaza H., Nonaka K., Kang D. (2013). Dihydro-orotate dehydrogenase is physically associated with the respiratory complex and its loss leads to mitochondrial dysfunction. Biosci. Rep..

[25] Rainger J., Bengani H., Campbell L., Anderson E., Sokhi K., Lam W., Riess A., Ansari M., Smithson S., Lees M. (2012). Miller (Genee-Wiedemann) syndrome represents a clinically and biochemically distinct subgroup of postaxial acrofacial dysostosis associated with partial deficiency of DHODH. Hum. Mol. Genet..

[26] Amos A., Amos A., Wu L., Xia H. (2023). The Warburg effect modulates DHODH role in ferroptosis: A review. Cell Commun. Signal.

[27] Reis R.A.G., Calil F.A., Feliciano P.R., Pinheiro M.P., Nonato M.C. (2017). The dihydroorotate dehydrogenases: Past and present. Arch. Biochem. Biophys..

[28] Banerjee R., Purhonen J., Kallijärvi J. (2022). The mitochondrial coenzyme Q junction and complex III: Biochemistry and pathophysiology. FEBS J..

[29] Mercola J. (2025). Reductive stress and mitochondrial dysfunction: The hidden link in chronic disease. Free Radic. Biol. Med..

[30] Chen B., Lyssiotis C.A., Shah Y.M. (2025). Mitochondria-organelle crosstalk in establishing compartmentalized metabolic homeostasis. Mol. Cell.

[31] Orozco Rodriguez J.M., Wacklin-Knecht H.P., Clifton L.A., Bogojevic O., Leung A., Fragneto G., Knecht W. (2022). New Insights into the Interaction of Class II Dihydroorotate Dehydrogenases with Ubiquinone in Lipid Bilayers as a Function of Lipid Composition. Int. J. Mol. Sci..

[32] Ullrich A., Knecht W., Piskur J., Löffler M. (2002). Plant dihydroorotate dehydrogenase differs significantly in substrate specificity and inhibition from the animal enzymes. FEBS Lett..

[33] de Mori R.M., Aleixo M.A.A., Zapata L.C.C., Calil F.A., Emery F.S., Nonato M.C. (2021). Structural basis for the function and inhibition of dihydroorotate dehydrogenase from Schistosoma mansoni. FEBS J..

[34] Jarmuszkiewicz W., Dominiak K., Budzinska A., Wojcicki K., Galganski L. (2023). Mitochondrial Coenzyme Q Redox Homeostasis and Reactive Oxygen Species Production. Front. Biosci. (Landmark Ed).

[35] Agarwal A.A., Georgiades J.D., Dranow D.M., Lorimer D.D., Edwards T., Barrett K.F., Craig J.K., Van Voorhis W.C., Myler P.J., Smith C.L. (2025). Crystal structure of dihydroorotate dehydrogenase from Helicobacter pylori with bound flavin mononucleotide. Acta Crystallogr. F Struct. Biol. Commun..

[36] Chen J., Yang S., Li Y., Ziwen X., Zhang P., Song Q., Yao Y., Pei H. (2023). De novo nucleotide biosynthetic pathway and cancer. Genes Dis..

[37] Bajzikova M., Kovarova J., Coelho A.R., Boukalova S., Oh S., Rohlenova K., Svec D., Hubackova S., Endaya B., Judasova K. (2019). Reactivation of Dihydroorotate Dehydrogenase-Driven Pyrimidine Biosynthesis Restores Tumor Growth of Respiration-Deficient Cancer Cells. Cell Metab..

[38] So J., Lewis A.C., Smith L.K., Stanley K., Franich R., Yoannidis D., Pijpers L., Dominguez P., Hogg S.J., Vervoort S.J. (2022). Inhibition of pyrimidine biosynthesis targets protein translation in acute myeloid leukemia. EMBO Mol. Med..

[39] Rhaman M.M., Powell D.R., Hossain M.A. (2017). Supramolecular Assembly of Uridine Monophosphate (UMP) and Thymidine Monophosphate (TMP) with a Dinuclear Copper(II) Receptor. ACS Omega.

[40] Shi D.D., Savani M.R., Levitt M.M., Wang A.C., Endress J.E., Bird C.E., Buehler J., Stopka S.A., Regan M.S., Lin Y.F. (2022). De novo pyrimidine synthesis is a targetable vulnerability in IDH mutant glioma. Cancer Cell.

[41] Diehl F.F., Miettinen T.P., Elbashir R., Nabel C.S., Darnell A.M., Do B.T., Manalis S.R., Lewis C.A., Vander Heiden M.G. (2022). Nucleotide imbalance decouples cell growth from cell proliferation. Nat. Cell Biol..

[42] Zhou Y., Tao L., Zhou X., Zuo Z., Gong J., Liu X., Zhou Y., Liu C., Sang N., Liu H. (2021). DHODH and cancer: Promising prospects to be explored. Cancer Metab..

[43] Zhang L., Zhang J., Wang J., Ren C., Tang P., Ouyang L., Wang Y. (2022). Recent advances of human dihydroorotate dehydrogenase inhibitors for cancer therapy: Current development and future perspectives. Eur. J. Med. Chem..

[44] Wang Y., Lilienfeldt N., Hekimi S. (2024). Understanding coenzyme Q. Physiol. Rev..

[45] Li J., Jia Y.C., Ding Y.X., Bai J., Cao F., Li F. (2023). The crosstalk between ferroptosis and mitochondrial dynamic regulatory networks. Int. J. Biol. Sci..

[46] Hai Y., Fan R., Zhao T., Lin R., Zhuang J., Deng A., Meng S., Hou Z., Wei G. (2024). A novel mitochondria-targeting DHODH inhibitor induces robust ferroptosis and alleviates immune suppression. Pharmacol. Res..

[47] Junco M., Ventura C., Santiago Valtierra F.X., Maldonado E.N. (2024). Facts, Dogmas, and Unknowns About Mitochondrial Reactive Oxygen Species in Cancer. Antioxidants.

[48] Pavlova N.N., Zhu J., Thompson C.B. (2022). The hallmarks of cancer metabolism: Still emerging. Cell Metab..

[49] Yang C., Zhao Y., Wang L., Guo Z., Ma L., Yang R., Wu Y., Li X., Niu J., Chu Q. (2023). De novo pyrimidine biosynthetic complexes support cancer cell proliferation and ferroptosis defence. Nat. Cell Biol..

[50] Zhang H., Santana-Codina N., Yu Q., Poupault C., Campos C., Qin X., Sindoni N., Ciscar M., Padhye A., Kuljanin M. (2025). De novo pyrimidine biosynthesis inhibition synergizes with BCL-X(L) targeting in pancreatic cancer. Nat. Commun..

[51] Zhang Q., Cui K., Kong Y., Yu J., Luo Z., Yang X., Gong L., Xie Y., Lin J., Liu C. (2025). Targeting both the enzymatic and non-enzymatic functions of DHODH as a therapeutic vulnerability in c-Myc-driven cancer. Cell Rep..

[52] Qian Y., Liang X., Kong P., Cheng Y., Cui H., Yan T., Wang J., Zhang L., Liu Y., Guo S. (2020). Elevated DHODH expression promotes cell proliferation via stabilizing β-catenin in esophageal squamous cell carcinoma. Cell Death Dis..

[53] Wu D., Wang W., Chen W., Lian F., Lang L., Huang Y., Xu Y., Zhang N., Chen Y., Liu M. (2018). Pharmacological inhibition of dihydroorotate dehydrogenase induces apoptosis and differentiation in acute myeloid leukemia cells. Haematologica.

[54] Shi Z.Z., Jin X., Li W.T., Tao H., Song S.J., Fan Z.W., Jiang W., Liang J.W., Bai J. (2023). Dihydroorotate dehydrogenase promotes cell proliferation and suppresses cell death in esophageal squamous cell carcinoma and colorectal carcinoma. Transl. Cancer Res..

[55] Xie Y., Kang R., Klionsky D.J., Tang D. (2023). GPX4 in cell death, autophagy, and disease. Autophagy.

[56] Lee J., Roh J.L. (2023). Targeting GPX4 in human cancer: Implications of ferroptosis induction for tackling cancer resilience. Cancer Lett..

[57] Lee J., Roh J.L. (2022). SLC7A11 as a Gateway of Metabolic Perturbation and Ferroptosis Vulnerability in Cancer. Antioxidants.

[58] Jiang X., Stockwell B.R., Conrad M. (2021). Ferroptosis: Mechanisms, biology and role in disease. Nat. Rev. Mol. Cell Biol..

[59] Bersuker K., Hendricks J.M., Li Z., Magtanong L., Ford B., Tang P.H., Roberts M.A., Tong B., Maimone T.J., Zoncu R. (2019). The CoQ oxidoreductase FSP1 acts parallel to GPX4 to inhibit ferroptosis. Nature.

[60] Doll S., Freitas F.P., Shah R., Aldrovandi M., da Silva M.C., Ingold I., Goya Grocin A., Xavier da Silva T.N., Panzilius E., Scheel C.H. (2019). FSP1 is a glutathione-independent ferroptosis suppressor. Nature.

[61] Jin D.Y., Chen X., Liu Y., Williams C.M., Pedersen L.C., Stafford D.W., Tie J.K. (2023). A genome-wide CRISPR-Cas9 knockout screen identifies FSP1 as the warfarin-resistant vitamin K reductase. Nat. Commun..

[62] Gan B. (2021). Mitochondrial regulation of ferroptosis. J. Cell Biol..

[63] Pang Q., Tang Z., Luo L. (2024). The crosstalk between oncogenic signaling and ferroptosis in cancer. Crit. Rev. Oncol. Hematol..

[64] Berndt C., Alborzinia H., Amen V.S., Ayton S., Barayeu U., Bartelt A., Bayir H., Bebber C.M., Birsoy K., Böttcher J.P. (2024). Ferroptosis in health and disease. Redox Biol..

[65] Yuan Z., Wang X., Qin B., Hu R., Miao R., Zhou Y., Wang L., Liu T. (2024). Targeting NQO1 induces ferroptosis and triggers anti-tumor immunity in immunotherapy-resistant KEAP1-deficient cancers. Drug Resist. Updat..

[66] Hayes J.D., Dinkova-Kostova A.T., Tew K.D. (2020). Oxidative Stress in Cancer. Cancer Cell.

[67] Mailloux R.J. (2024). Proline and dihydroorotate dehydrogenase promote a hyper-proliferative state and dampen ferroptosis in cancer cells by rewiring mitochondrial redox metabolism. Biochim. Biophys. Acta Mol. Cell Res..

[68] Jiang M., Song Y., Liu H., Jin Y., Li R., Zhu X. (2023). DHODH Inhibition Exerts Synergistic Therapeutic Effect with Cisplatin to Induce Ferroptosis in Cervical Cancer through Regulating mTOR Pathway. Cancers.

[69] Hubackova S., Davidova E., Boukalova S., Kovarova J., Bajzikova M., Coelho A., Terp M.G., Ditzel H.J., Rohlena J., Neuzil J. (2020). Replication and ribosomal stress induced by targeting pyrimidine synthesis and cellular checkpoints suppress p53-deficient tumors. Cell Death Dis..

[70] Lafita-Navarro M.C., Venkateswaran N., Kilgore J.A., Kanji S., Han J., Barnes S., Williams N.S., Buszczak M., Burma S., Conacci-Sorrell M. (2020). Inhibition of the de novo pyrimidine biosynthesis pathway limits ribosomal RNA transcription causing nucleolar stress in glioblastoma cells. PLoS Genet..

[71] Olsen T.K., Dyberg C., Embaie B.T., Alchahin A., Milosevic J., Ding J., Otte J., Tümmler C., Hed Myrberg I., Westerhout E.M. (2022). DHODH is an independent prognostic marker and potent therapeutic target in neuroblastoma. JCI Insight.

[72] Yamaguchi N., Weinberg E.M., Nguyen A., Liberti M.V., Goodarzi H., Janjigian Y.Y., Paty P.B., Saltz L.B., Kingham T.P., Loo J.M. (2019). PCK1 and DHODH drive colorectal cancer liver metastatic colonization and hypoxic growth by promoting nucleotide synthesis. Elife.

[73] Gao W., Hu L., Zhang M., Liu S., Xu S., Chow V.L., Chan J.Y., Wong T.S. (2022). Mitochondrial DHODH regulates hypoxia-inducible factor 1 expression in OTSCC. Am. J. Cancer Res..

[74] Li O., An K., Wang H., Li X., Wang Y., Huang L., Du Y., Qin N., Dong J., Wei J. (2025). Targeting YBX1-m5C mediates RNF115 mRNA circularisation and translation to enhance vulnerability of ferroptosis in hepatocellular carcinoma. Clin. Transl. Med..

[75] Amos A., Jiang N., Zong D., Gu J., Zhou J., Yin L., He X., Xu Y., Wu L. (2023). Depletion of SOD2 enhances nasopharyngeal carcinoma cell radiosensitivity via ferroptosis induction modulated by DHODH inhibition. BMC Cancer.

[76] Ding Q., Tang W., Li X., Ding Y., Chen X., Cao W., Wang X., Mo W., Su Z., Zhang Q. (2023). Mitochondrial-targeted brequinar liposome boosted mitochondrial-related ferroptosis for promoting checkpoint blockade immunotherapy in bladder cancer. J. Control. Release.

[77] Ishikawa C., Mori N. (2024). Pivotal role of dihydroorotate dehydrogenase as a therapeutic target in adult T-cell leukemia. Eur. J. Haematol..

[78] Hosseini M., Dousset L., Michon P., Mahfouf W., Muzotte E., Bergeron V., Bortolotto D., Rossignol R., Moisan F., Taieb A. (2019). UVB-induced DHODH upregulation, which is driven by STAT3, is a promising target for chemoprevention and combination therapy of photocarcinogenesis. Oncogenesis.

[79] Madak J.T., Bankhead A., Cuthbertson C.R., Showalter H.D., Neamati N. (2019). Revisiting the role of dihydroorotate dehydrogenase as a therapeutic target for cancer. Pharmacol. Ther..

[80] Zhan M., Ding Y., Huang S., Liu Y., Xiao J., Yu H., Lu L., Wang X. (2023). Lysyl oxidase-like 3 restrains mitochondrial ferroptosis to promote liver cancer chemoresistance by stabilizing dihydroorotate dehydrogenase. Nat. Commun..

[81] Li C., Zhou Y., Xu J., Zhou X., Huang Z., Zeng T., Yang X., Tao L., Gou K., Zhong X. (2022). A novel series of teriflunomide derivatives as orally active inhibitors of human dihydroorotate dehydrogenase for the treatment of colorectal carcinoma. Eur. J. Med. Chem..

[82] Mullen N.J., Shukla S.K., Thakur R., Kollala S.S., Wang D., Chaika N., Santana J.F., Miklavcic W.R., LaBreck D.A., Mallareddy J.R. (2024). DHODH inhibition enhances the efficacy of immune checkpoint blockade by increasing cancer cell antigen presentation. Elife.

[83] Zhang J.F., Hong L.H., Fan S.Y., Zhu L., Yu Z.P., Chen C., Kong L.Y., Luo J.G. (2024). Discovery of piperine derivatives as inhibitors of human dihydroorotate dehydrogenase to induce ferroptosis in cancer cells. Bioorg Chem..

[84] Christian S., Merz C., Evans L., Gradl S., Seidel H., Friberg A., Eheim A., Lejeune P., Brzezinka K., Zimmermann K. (2019). The novel dihydroorotate dehydrogenase (DHODH) inhibitor BAY 2402234 triggers differentiation and is effective in the treatment of myeloid malignancies. Leukemia.

[85] Wang F., Min J. (2021). DHODH tangoing with GPX4 on the ferroptotic stage. Signal Transduct. Target. Ther..

[86] Zhou T.J., Zhang M.M., Liu D.M., Huang L.L., Yu H.Q., Wang Y., Xing L., Jiang H.L. (2024). Glutathione depletion and dihydroorotate dehydrogenase inhibition actuated ferroptosis-augment to surmount triple-negative breast cancer. Biomaterials.

[87] Yao L., Yang N., Zhou W., Akhtar M.H., Zhou W., Liu C., Song S., Li Y., Han W., Yu C. (2023). Exploiting Cancer Vulnerabilities by Blocking of the DHODH and GPX4 Pathways: A Multifunctional Bodipy/PROTAC Nanoplatform for the Efficient Synergistic Ferroptosis Therapy. Adv. Healthc. Mater..

[88] Mishima E., Nakamura T., Zheng J., Zhang W., Mourão A.S.D., Sennhenn P., Conrad M. (2023). DHODH inhibitors sensitize to ferroptosis by FSP1 inhibition. Nature.

[89] Mathur D., Stratikopoulos E., Ozturk S., Steinbach N., Pegno S., Schoenfeld S., Yong R., Murty V.V., Asara J.M., Cantley L.C. (2017). PTEN Regulates Glutamine Flux to Pyrimidine Synthesis and Sensitivity to Dihydroorotate Dehydrogenase Inhibition. Cancer Discov..

[90] Chen S., Yang J., Liang Z., Li Z., Xiong W., Fan Q., Shen Z., Liu J., Xu Y. (2023). Synergistic Functional Nanomedicine Enhances Ferroptosis Therapy for Breast Tumors by a Blocking Defensive Redox System. ACS Appl. Mater. Interfaces.

[91] Xia Y., Sun M., Huang H., Jin W.L. (2024). Drug repurposing for cancer therapy. Signal Transduct. Target. Ther..

[92] Zhang Z., Liu X., Chen D., Yu J. (2022). Radiotherapy combined with immunotherapy: The dawn of cancer treatment. Signal Transduct. Target. Ther..

[93] Liang Y., Zhao Y., Qi Z., Li X., Zhao Y. (2025). Ferroptosis: CD8(+)T cells’ blade to destroy tumor cells or poison for self-destruction. Cell Death Discov..

[94] Alsaafeen B.H., Ali B.R., Elkord E. (2025). Resistance mechanisms to immune checkpoint inhibitors: Updated insights. Mol. Cancer.

[95] Lee J.B., Kim H.R., Ha S.J. (2022). Immune Checkpoint Inhibitors in 10 Years: Contribution of Basic Research and Clinical Application in Cancer Immunotherapy. Immune Netw..

[96] Ebrahimnezhad M., Valizadeh A., Yousefi B. (2025). Ferroptosis and immunotherapy: Breaking barriers in cancer treatment resistance. Crit. Rev. Oncol. Hematol..

[97] Lei G., Zhuang L., Gan B. (2024). The roles of ferroptosis in cancer: Tumor suppression, tumor microenvironment, and therapeutic interventions. Cancer Cell.

[98] Cui K., Wang K., Huang Z. (2024). Ferroptosis and the tumor microenvironment. J. Exp. Clin. Cancer Res..

[99] Kay E.J., Zanivan S. (2025). The tumor microenvironment is an ecosystem sustained by metabolic interactions. Cell Rep..

[100] Arner E.N., Rathmell J.C. (2023). Metabolic programming and immune suppression in the tumor microenvironment. Cancer Cell.

[101] Zhang S., Kang L., Dai X., Chen J., Chen Z., Wang M., Jiang H., Wang X., Bu S., Liu X. (2022). Manganese induces tumor cell ferroptosis through type-I IFN dependent inhibition of mitochondrial dihydroorotate dehydrogenase. Free Radic. Biol. Med..

